# PDLIM1 inhibits cell migration and invasion in diabetic retinopathy via negatively regulating Wnt3a

**DOI:** 10.1038/s41598-023-33073-7

**Published:** 2023-04-10

**Authors:** Pinxue Xie, Qisheng You, Jiang Zhu, Wuxiang Xie, Ping Wei, Siquan Zhu, Yunhui Du, Xinxiao Gao

**Affiliations:** 1 Beijing Institute of Heart, Lung and Blood Vessel Diseases, Beijing, 100029 China; 2 Department of Ophthalmology, Beijing Anzhen Hospital, Capital Medical University, Beijing, 100029 China; 3 Kresge Eye Institute, Wayne State University, Detroit, MI USA; 4 Key Laboratory of the Ministry of Education for Optoelectronic Measurement Technology and Instrument, Beijing Information Science and Technology University, Beijing, 100192 China; 5 Peking University Clinical Research Institute, Peking University, Beijing, 100029 China; 6 Casey Eye Institute, Oregon Health & Science University, Portland, OR 97239 USA; 7 Beijing Key Laboratory of Upper Airway Dysfunction-Related Cardiovascular Diseases, Beijing Institute of Heart, Lung and Blood Vessel Diseases, Beijing Anzhen Hospital, Capital Medical University, Beijing, 100029 China

**Keywords:** Cell biology, Pathogenesis

## Abstract

The injury of vascular endothelial cells is a crucial factor in the development of diabetic retinopathy (DR). PDLIM1 (a member of the PDZ and LIM protein family) has been reported to exert an essential function in vascular diseases. This study aimed to elucidate the role of PDLIM1 on retinal vascular endothelial cells in DR. Immunofluorescence staining was used to localize the expression of PDLIM1 in the mouse retina. In some tumor diseases, PDLIM1 has been reported to play a key role in regulating the Wnt pathway. However, no in-depth reports have been found in DR. Retinal capillary endothelial cells (RCECs) were treated with high-glucose and high-lipid (HG/HL) culture medium, and siRNA transfection to investigate the role of PDLIM1 in DR. PDLIM1 and Wnt3a expression was confirmed by qRT-PCR and western blotting. Flow cytometry, Transwell assay, and scratch assay were used to test the ability of cell apoptosis, migration, and invasion. PDLIM1 was mainly expressed in the retinal pigment epithelium (RPE), ganglion cell layer (GCL), inner plexus layer (IPL), and outer plexus layer (OPL). HG/HL increased Wnt3a levels and promoted cell’s ability of apoptosis, migration, and invasion, which were reversed by the knockdown of PDLIM1. PDLIM1 was found to play a protective role in diabetic retinopathy by counter-regulating Wnt3a. PDLIM1 ameliorates cell apoptosis, migration, and invasion by negatively regulating Wnt3a in RCECs of DR, which suggests that PDLIM1 might be a promising therapeutic target for DR treatment.

## Introduction

With the aging of the population, the global diabetes mellitus (DM) prevalence will increase to 642.1 million by 2040^1^. Diabetic retinopathy (DR) is one of the most common complications of DM, it is estimated to occur in 75% of type 1 diabetes, and there are also 50% of type 2 diabetes will occur DR^2^. The progression of DR may seriously affect the vision of these patients and even cause blindness.

Up to today, the pathogenesis of DR is still not fully understood. It's suggested that the onset and progression of DR may be associated with abnormalities in polyol-inositol metabolism, protein kinase activation, formation of advanced glycosylation end products (AGEs), oxidative stress, and increases in inflammatory factors and vascular endothelial growth factor (VEGF)^3^. VEGF has been demonstrated to play an essential role in the development of DR by many studies^4–6^. As a result, anti-VEGF therapy has been widely used in recent years and effectively improves the long-term vision of DR patients^7^. However, anti-VEGF therapy has limitations, such as requiring multiple intravitreal injections, which may increase the infection risk and cause social and economic burdens to patients. In addition, some patients may respond poorly to the therapy.

PDLIM1 (a member of the PDZ and LIM protein family) is a structural protein in cell membranes and is widely distributed in heart, lung, liver and spleen tissues, and is expressed in human endothelial cells^8,9^. PDLIM1 is involved in the regulation of multiple protein pathways and plays a critical role in the development of tumors, including breast, ovarian, colorectal, liver, glioma and other cancers^10–12^. In chronic myeloid leukemia, PDLIM1 has been reported as a target to promote the migration of cancer cells via the Wnt/β-catenin pathway^13,14^.

In the study by Wan et al., microRNA-200a was found to have a protective effect in diabetic retinopathy by down-regulating PDLIM1. In their study, it was found that miR-200a treatment could significantly inhibit retinal permeability and inflammatory factors^15^. Our previous study showed that the levels of Wnt3a and VEGF in vitreous were increased and correlated with each other in proliferative diabetic retinopathy (PDR) patients^16^. Both Wnt3a and β-catenin play an important role in the Wnt signaling pathway, regulating cellular inflammation-related effects^17^. Therefore, Wnt3a may be an important role for DR, and its vitreous activity may be a biomarker of PDR^16^. According to previous reports, PDLIM1 has a regulatory effect on the Wnt/β-catenin pathway in colorectal cancer and chronic myeloid leukemia^13,14^, whereas its detailed function in DR has not been reported. In this study, we aim to investigate the role of PDLIM1 in DR by high-glucose and high-lipid(HG/HL) culture of RCECs and siRNA knockdown.

## Methods and materials

### Diabetic mouse model and animal ethics

All animal experiments of this study were performed in adherence to the ARRIVE guidelines. And all animal handling complied with the standard animal welfare regulations of Capital Medical University (Beijing, China). The Animal Subjects Committee of Capital Medical University approved the animal study protocol (No. 202104930). All animal anesthesia and euthanasia procedures comply with the American Veterinary Medical Association (AVMA) Guidelines. Male homozygous db/db mice (C57BKS-Lepr/db/db/JOrlRj) and C57BL/6J mice were purchased from Nanjing Junke Bioengineering Corporation, Ltd (Nanjing City, Jiangsu Province, China). Mice are housed under standard conditions for lighting (a 12 h/12 h light/dark cycle), temperature (23–25 °C) and humidity (50–60%), and water and food ad libitum for 16 weeks. Blood glucose levels were tested from the tail vein every two weeks. Animals were considered diabetic only if their blood sugar levels were more than three times as high as 16.7 mmol/L.

### Cell culture

Rat retinal capillary endothelial cells (RCECs) were purchased from Saibaikang (Shanghai, China). RCECs were cultured after 80% confluence in either normal glucose/normal lipid (NG/NL) or high glucose (4.5 g/L)/high lipidt (HG/HL) endothelial cell medium (ScienCell, America) containing 25 mM d-glucose and 250 μM palmitate. Both media contained 10% fetal bovine serum (FBS) (Gibco, America) and endothelial growth factor (ScienCell, America) according to the instruction. Then RCECs were inoculated in 5% Gelatin-coated cell culture flasks and placed in a 37 ℃, 5% CO_2_ incubator. The isolated RCECs were identified using the endothelial cell-specific marker vWF (Proteintech, China) fluorescence staining.

### Immunofluorescence staining

The 16-week-old db/db mice were killed by intravenous anesthesia with excess pentobarbital sodium (100 mg/kg). The removed eyeballs were fixed overnight in 4% PFA at 4 °C, immersed in 15% sucrose, followed by 30% sucrose in PBS, and finally frozen for storage at − 80 °C. Frozen sections were incubated in 95 °C citrate buffer (pH 6.0) for 30 min and then cooled to room temperature for next step. Sections were incubated overnight with 5% NDs (normal donkey serum, Jackson ImmunoResearch, America) at 4 °C at a 1:100 dilution of Wnt3a (abcam, ab219412) and PDLIM1 (abcam, ab129015). After rinsing with PBS-Tween (1:2000; Sigma-Aldrich), the cells were incubated with Alexa fluoroconjugated secondary antibody for 1 h. DAPI was used to counterstain the nucleus. Fluorescent images were obtained with ZEISS LSM880 confocal microscope (Carl Zeiss). Semi-quantification of immunofluorescence mainly used Image J software (National Institutes of Health, America) for semi-quantitative analysis of protein fluorescence expression intensity and statistical description.

### Flow cytometry assays

The cell density of RCECs was adjusted to 2 × 10^5^ cells/mL with DMEM complete medium, then inoculated into 24-well cell culture plates (500 μL per well). SiPDLIM1 transfection was started when the cell confluence reached 70%. Transfection was performed using Lipofectamine 3000 (Thermo Fisher, America) according to the protocol. Six hours after adding the transfection reagent, the medium in the culture wells was aspirated, discarded, and replaced with NG/NL concentration DMEM (5 mM) and HG/HL DMEM (25 mM) complete medium. The cells were harvested 24 and 72 h respectively after medium replacement, and flow cytometry analyzed apoptosis in each group. (BeamCyte, China).

### Wound-healing assay

Drawn with a marker on a 6-well plate of RCECs in the logarithmic growth phase. 5 × 10^5^ cells were added to each well to spread overnight, and the next day the cells were scratched with a sterile tip following the marker pen marks. Petri dishes were washed with PBS to remove floating cells and incubated at 37 °C in a 5% CO_2_ incubator and photographed at 0 and 48 h. The percentage of wound healing was measured using Image J software (National Institutes of Health, America).

### Transwell assay

The cell density was adjusted to 1 × 10^5^/mL in serum-free medium with 0.1% BSA in a 24-well plate with 500 μL of DMEM. After adding 200 μL of cell suspension inside the Transwell, the chambers were carefully transferred to the 24-well plate containing DMEM and then placed in a cell incubator (5% CO_2_, 37 °C) for 24 h. The medium of the upper chamber was gently aspirated, and the bottom surface of the chamber was immersed in 10% methanol solution for 30 s to fix the cells, transferred to pure water, and washed. Then the bottom surface of the chamber was instilled with crystal violet staining for 2 min and washed with pure water until the background was clear for observation.

### RNA extraction and quantitative PCR assay

Using Trizol^®^ (Thermo Fisher Scientific, MA, USA) extracted total RNA in RCECs, according to the manufacturer's instructions, and purified using SuperScript III RT Kit (Thermo Fisher Scientific, MA, USA). Purified RNA was quantified using NanoDrop Spectrophotometer (THERMO, USA) at a ratio of A260/A280. Each sample was then used for cDNA synthesis with 500 ng total RNA. Real-time qPCR was performed by using Applied Biosystem CFX1000 (Thermo Fisher Scientific, USA) and Sybrqpcr mix kit (Thermo Fisher Scientific, MA, USA). Expression of all genes was normalized to the housekeeping gene ACTIN. All PCR reactions were repeated three times, and the obtained data were analyzed by the 2^−ΔΔCt^ method.

The sequences of primers were as follows:

- PDLIM1: forward 5′-CTAGTGACCGAGGAGGGGAA-3′, reverse 5′-CGGTAGGGCTGTTGTACTGG′.
- Wnt3a: forward 5′-CTCCTCTCGGATACCTCTTAGTG-3′, reverse 5′-GCATGATCTCCACGTAGTTCCTG-3′.
- Actin: forward 5′-CCAGCCTTCCTTCTTGGGTA-3′, reverse 5′-CAATGCCTGGGTACATGGTG-3′.

### Western Blotting

RCECs were lysed with Radio Immunoprecipitation Assay (RIPA) buffer (Biyuntian, Shanghai, China). The cells were centrifuged at 14,000*g* for 20 min at 4 °C after ultrasonic shock by ice lysis, and the protein concentration was determined using the BCA protein assay (Biyuntian, Shanghai, China). Proteins were separated by dodecyl sulfate and sodium salt-polyacrylamide gel (SDS-PAGE) electrophoresis. Proteins of 30–50 µg were separated by 12.5% SDS–polyacrylamide gel electrophoresis. Total protein levels were determined with Wnt3a (abcam, ab219412), PDLIM1 (abcam, ab129015) and Actin (MDL, MDL11027) monoclonal rabbit antibodies (Cell Signaling Technology, USA). All antibodies were diluted 1:1000. After the application of the primary antibody, the PVDF (Millipore, Germany) was washed three times and then incubated with the secondary antibody, which was coupled with horseradish peroxidase and incubated for 2 h at 37 °C. The luminescence image analyzer Image J software (National Institutes of Health, America) was used to quantify the Western blot bands, and the optical densities of target genes were normalized by ACTIN.

### Statistical analyses

GraphPad Prism 7 (GraphPad Software, USA) was used to all statistical analyses and graph plotting in our study. And measurement data in our study were analyzed using the t-test or one-way ANOVA. Less than 0.05 of P-value was considered significant.

## Results

### PDLIM1 expression decreases in vitro and in vivo in DR

**Figure 1:**
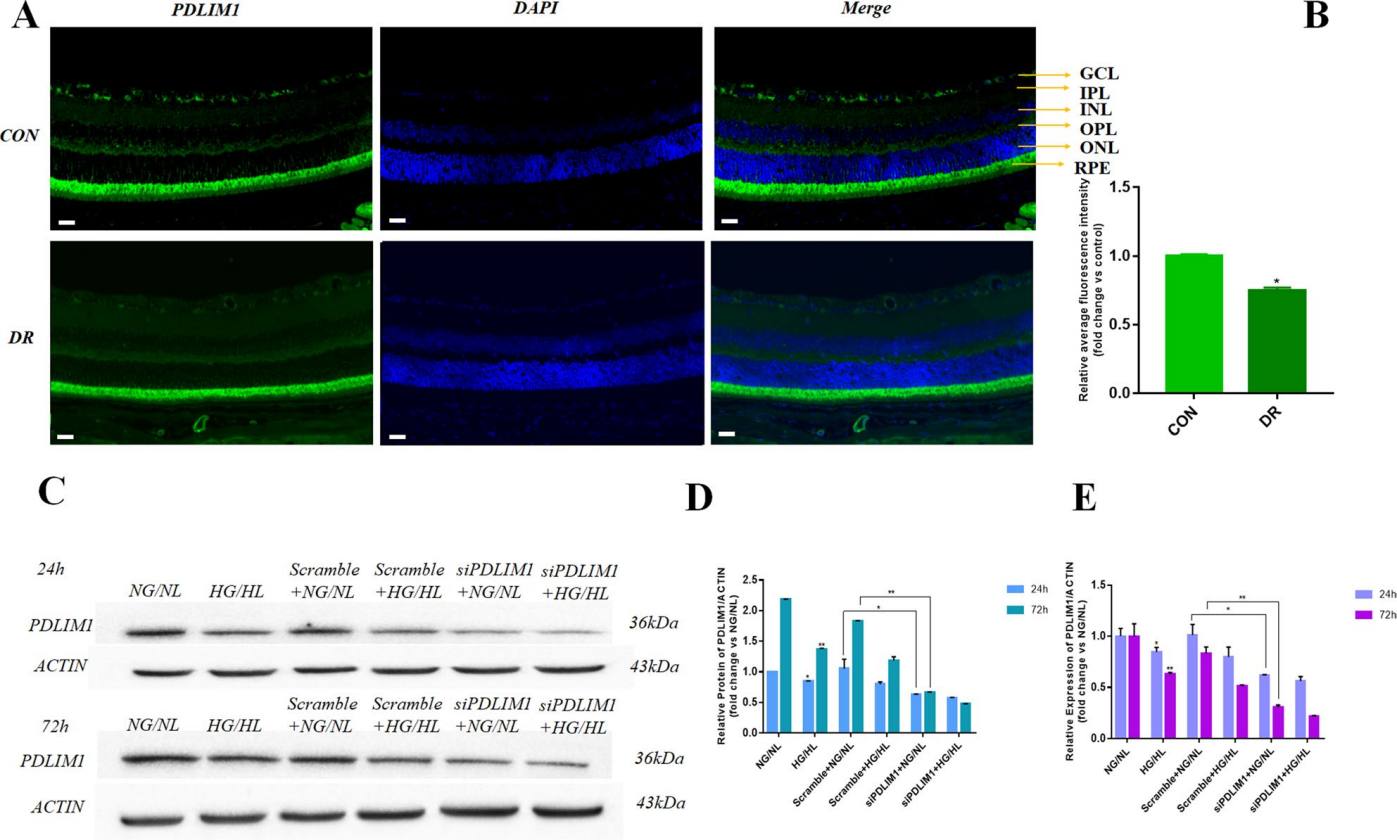
The expression of PDLIM1 in mouse retina and retinal capillary endothelial cells (RCECs). (**A**) Immunofluorescence staining shows that PDLIM1 is mainly expressed in the retinal pigment epithelium (RPE). Scale bar: 100 μm. (**B**) Semi-quantitative analysis showed that PDLIM1 expression decreased in DR mice compared to control. (**C**) Representative immunoblots show that the protein expression of PDLIM1 decreased after HG/HL treatment. (**D**) PDLIM1 decreased at 24 h in HG/HL (P < 0.05), and the trend was more evident after 72 h (P < 0.01) compared with those in NG/NL. (**E**) The mRNA expression of PDLIM1 downregulated at 24 h in HG/HL (P < 0.05), and the trend was more evident after 72 h (P < 0.01) compared with those in NG/NL. *P < 0.05, **P < 0.01. (*CON* control group, *DR* diabetic retinopathy, *GCL:* ganglion cell layer, *IPL:* inner plexiform layer, *INL:* inner nuclear layer, *OPL:* outer plexiform layer, *ONL:* outer nuclear layer, *RPE:* retinal pigment epithelium).

Immunofluorescence staining was performed on the retina of C57 mice. It was found that PDLIM1 was mainly expressed in the retinal pigment epithelium (RPE) layer. At the same time, a small amount of PDLIM1 was also expressed in the ganglion cell layer (GCL), inner plexus layer (IPL), and outer plexus layer (OPL) (Fig. 1A). The expression of PDLIM1 decreased in DR by immunofluorescence semi-quantitative comparison (P < 0.05, Fig. 1B). We further verified the PDLIM1 in RCECs by PCR and Western blotting. The protein and mRNA of PDLIM1 decreased at 24 h in HG/HL culture (P < 0.05, Fig. 1C–E), and the tendency was more evident after 72 h treatment (P < 0.01, Fig. 1C–E) compared with those in NG/NL. After 24 h of siPDLIM1 treatment, the expression of PDLIM1 protein and mRNA was significantly decreased compared with that of the scramble group, and the decrease was more obvious after 72 h of treatment. The reducing level of PDLIM1 in RCECs was consistent with the immunofluorescence results of mouse retinal cells.

### PDLIM1 negatively regulates the Wnt pathway in DR

**Figure 2:**
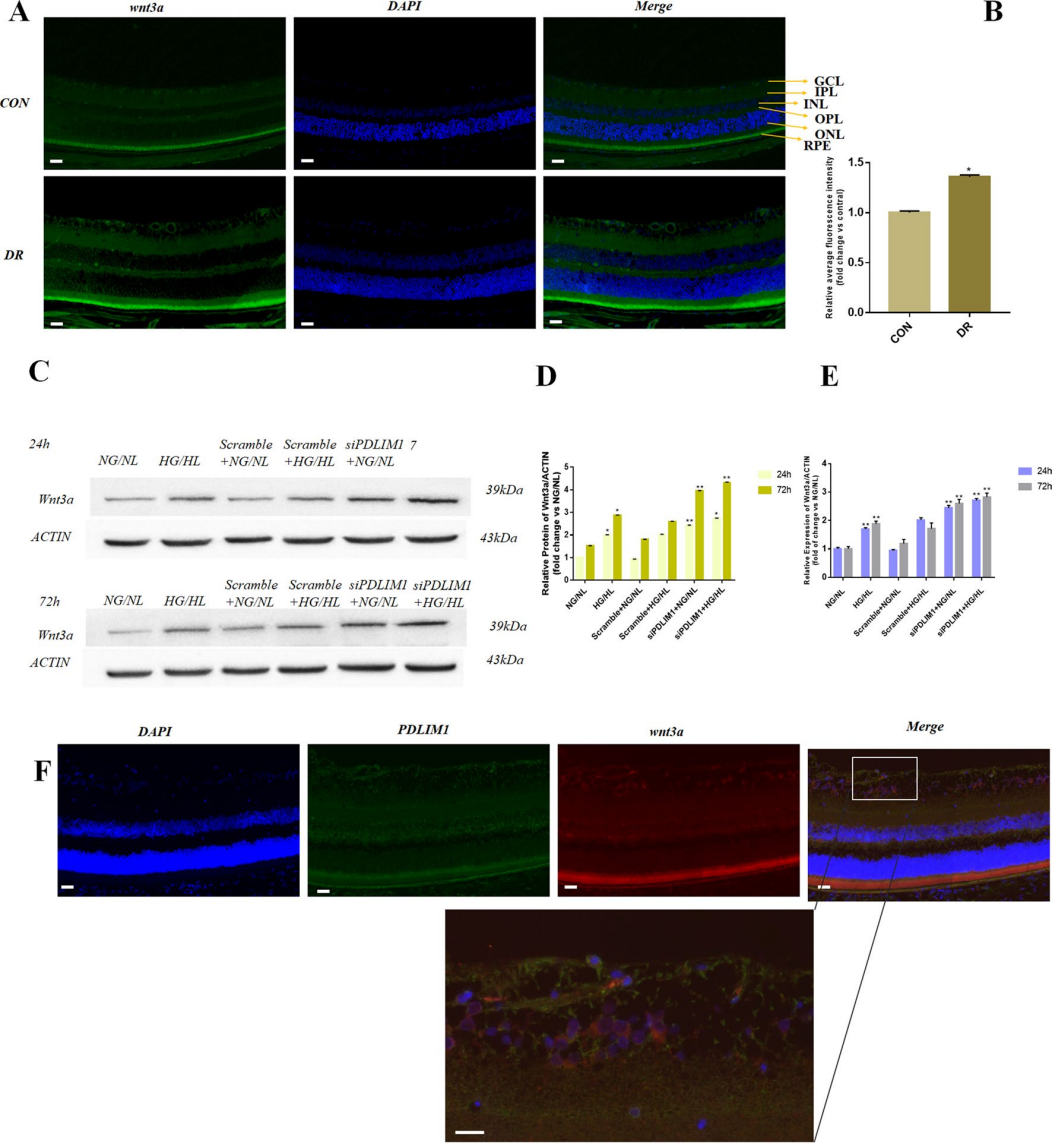
The expression of Wnt3a in mouse retina and retinal capillary endothelial cells (RCECs). (**A**) Immunofluorescence staining showed that Wnt3a was mainly expressed in the retinal pigment epithelium (RPE) layer, ganglion cell layer (GCL), the inner plexus (IPL) and outer plexus layer (OPL). Scale bar: 100 μm. (**B**) Semi-quantitative analysis showed that Wnt3a expression increased in DR mice. (**C**) Representative immunoblots showed that the protein expression of Wnt3a increased after HG/HL treatment and PDLIM1 knockdown. (**D**) Wnt3a protein level increased at 24 h/72 h in HG/HL compared with NG/NL. After PDLIM1 knockdown, the expression of Wnt3a increased at 24 h/72 h in RCECs, compared with scramble control. (**E**) The mRNA expression of Wnt3a upregulated at 24 h/72 h in HG/HL, compared with NG/NL. Wnt3a mRNA increased after PDLIM1 knockdown at 24 h/72 h, compared with scramble control. (**F**) Double staining of PDLIM1 and Wnt3a in the retina showed that the expression location of PDLIM1 and Wnt3a was basically the same, in which green was PDLIM1 and red was Wnt3a. (*CON:* control group, *DR:* diabetic retinopathy, *GCL:* ganglion cell layer, *IPL:* inner plexiform layer, *INL:* inner nuclear layer, *OPL:* outer plexiform layer, *ONL:* outer nuclear layer, *RPE:* retinal pigment epithelium). *P < 0.05, **P < 0.01.

Immunofluorescence staining showed that Wnt3a was mainly expressed in the RPE layer, GCL, IPL, and OPL, and the localization was consistent with PDLIM1 (Fig. 2A). Compared with control, the expression of Wnt3a increased in diabetic retinopathy mice measured by semi-quantitative analysis (P < 0.05, Fig. 2B). We performed double fluorescence staining of the retina for PDLIM1 and Wnt3a, and found that PDLIM1 and Wnt3a were expressed in basically the same location. (Fig. 2F) The results of double fluorescence staining can further indicate the relationship between PDLIM1 and Wnt3a. To investigate the changes of Wnt3a in DR and its relationship with PDLIM1, RCECs were treated in HG/HL with knockdown of PDLIM1. It showed that Wnt3a in RCECs increased in HG/HL compared with those in NG/NL (P < 0.01, Fig. 2C–E). In NG/NL culture, Wnt3a was also elevated in siPDLIM1-interfered RCECs, which was also supported that PDLIM1 reverse regulation of Wnt3a in RCECs (P < 0.01, Fig. 2C–E). At 72 h (P < 0.01), the Wnt3a level in HG/HL + siPDLIM1 group increased more significantly than that at 24 h (P < 0.05). These results indicated that under HG/HL culture conditions, as long as siPDLIM1 was treated for a long time, it could further increase the expression of Wnt3a by regulating the Wnt pathway. After being treated with siPDLIM1, the upregulated expression of Wnt3a was more prominent, which indicates that PDLIM1 negatively regulates the expression of Wnt3a in DR.

### PDLIM1 plays a vascular protective role in DR

**Figure 3:**
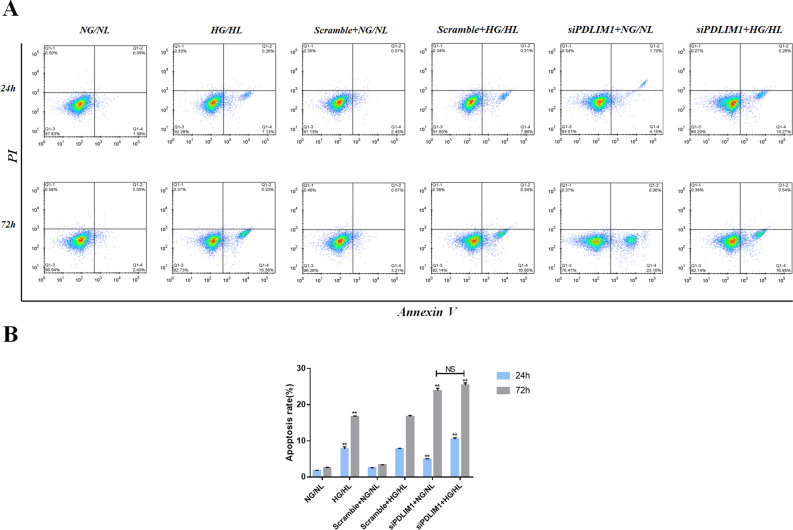
PDLIM1 reduces apoptosis of retinal capillary endothelial cells (RCECs) in DR. (**A**) Cell apoptosis rate was detected by flow cytometry. (**B**) At 24 h/72 h, apoptosis increased after HG/HL treatment, compared with NG/NL. Apoptosis of RCECs increased after PDLIM1 knockdown compared with Scramble control. After 72 h of PDLIM1 knockdown, RCECs were significantly apoptotic. *P < 0.05, **P < 0.01; NS, no significance.

**Figure 4:**
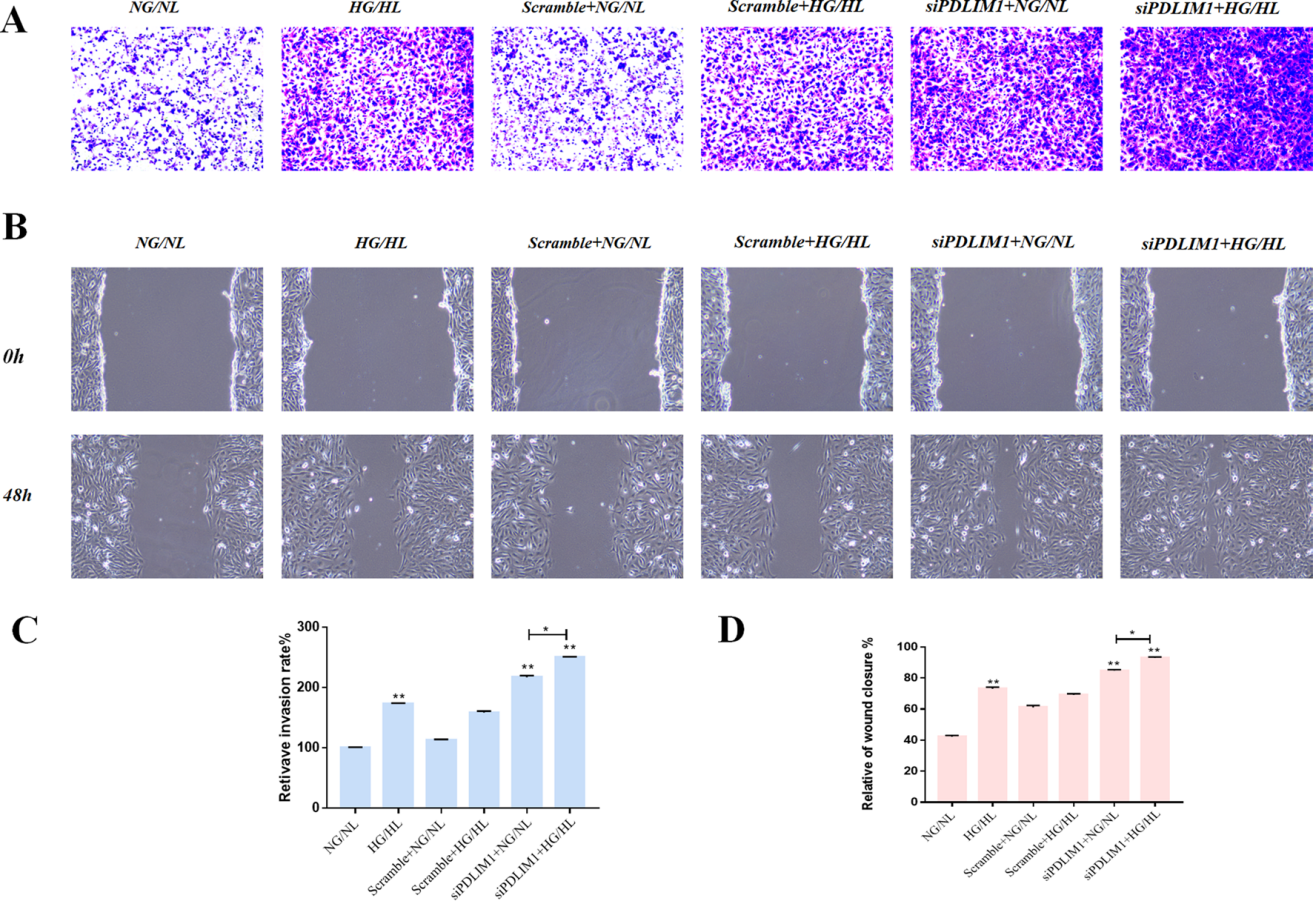
PDLIM1 inhibits the invasion and migration of retinal capillary endothelial cells (RCECs). (**A**) Transwell was used to compare the invasion of RCECs 24 h after HG/HL and PDLIM1 transfection. Scale bar: 50 μm (**B**) The migration of RCECs 48 h after HG/HL and PDLIM1 transfection was compared by scratch test. Scale bar: 50 μm. (**C**) The invasion of cells increased after HG/HL treatment compared with NG/NL. The invasion of RCECs was enhanced after PDLIM1 knockdown, compared with Scramble control. (**D**) The migration of cells increased after HG/HL treatment compared with NG/NL. The migration of RCECs was promoted after PDLIM1 knockdown, compared with Scramble control. **P < 0.01.

To explore the effect of PDLIM1 on vascular function in DR, flow cytometry assays, Transwell assay, and scratch tests were performed on RCECs with knockdown of PDLIM1 in HG/HL culture. Our results showed that HG/HL treatment significantly increased apoptosis of RCECs compared with NG/NL culture at 24 h and 72 h (P1 < 0.01; P2 < 0.01, Fig. 3). Apoptosis was significantly increased after knockdown of PDLIM1, while the apoptosis rate was more significantly increased in the group of siPDLIM1 + HG/HL (P < 0.01, Fig. 3). We found that at 72 h, the same apoptosis rate was observed in both NG/NL + siPDLIM1 and HG/HL + siPDLIM1 (P = NS). We believe that this may be because siPDLIM1 began to decline in cell state after 72 h of interference with RCECs, so the same apoptotic manifestations appeared at 72 h. In order to study the effect of PDLIM1 on the invasion ability of RCECs in DR, we first evaluated the effect of HG/HL on the invasion ability of RCECs. Transwell experiment showed that HG/HL increased the invasion ability of RCECs compared to NG/NL (P < 0.01, Fig. 4A,C). Furthermore, our results showed that knockdown of PDLIM1 significantly enhanced the invasion ability of RCECs cells when compared with the scramble control cells, both in HG/HL and NG/NL culture (P < 0.01, Fig. 4A,C). These findings suggested that PDLIM1 could effectively reduce the invasion of RCECs cells in DR. Then, the scratch test was used to study the effect of PDLIM1 on the migration of RCECs in DR. We found that the HG/HL significantly increased the migration of RCECs compared to NG/NL (P < 0.01, Fig. 4B,D). Moreover, our results showed that knockdown of PDLIM1 markedly increased the migration ability of RCECs cells when compared with the scramble control cells, both in HG/HL and NG/NL culture (P < 0.01, Fig. 4B,D). These data indicated that HG/HL allowed or enhanced the migration of RCECs cells after the depression of PDLIM1.

## Discussion

Previous studies have suggested that PDLIM1 is crucial in various physiological conditions and cell proliferation and metastasis during tumor progression^14^. However, till now, the studies on PDLIM1 in ocular diseases are mainly lacking. By observing chicken retinas, Hugo et al.^18^ found that PDLIM1 primarily localizes in the inner and outer plexus layer, the outer edge layer of muller cells, and photoreceptor cells. Their results showed that this protein also exists in the cone pedicle and other synapses of chicken retinas^18^. Similarly, our immunofluorescence staining suggested that PDLIM1 was mainly located in the RPE in the mouse retina. A small amount of PDLIM1 was also expressed in the GCL, IPL, and OPL. Semi-quantitative results showed that the expression of PDLIM1 decreased in DR compared to controls. Meanwhile, we double-stained one retinal section for PDLIM1 and Wnt3a. The results showed that the expression location of PDLIM1 and Wnt3a in the retina was basically the same. This further indicated that PDLIM1 may have a regulatory effect on Wnt3a. Moreover, we also identified the decreased expression of PDLIM1 in RCECs after HG/HL treatment in vitro experiments. All these results indicate that PDLIM1 may be crucial in the pathogenesis of DR.

PDLIM1 has been shown to regulate β-catenin in the classic Wnt pathway in colorectal cancer and promote the migration of cancer cells in chronic myeloid leukemia through the Wnt/β-catenin pathway^13,14^. In addition, Chen et al.^17^ found that the upregulated expression of Wnt/β-catenin in bone cells can enhance the proliferation and metabolism of bone cells in diabetes. In a previous study, Wan et al. found that of PDLIM1 in the development of DR is significant. In their study, human retinal microvascular endothelial cells (HRMECs) were treated with high glucose and miR-200A, and mice were intraperitoneally injected with STZ to induce diabetes, which is similar to a model of type 1 diabetes^15,19^. In order to get closer to the physiological environment and avoid the tumor-cell nature of the cell line, primary rat retinal capillary endothelial cells were used in our study^20^. And unlike Wan’s study, db/db mice were cultured for animal model, and high-glucose/high-lipids were used for cell culture in current study. Db/db mice were high-glucose and high-fat mice with congenital leptin deficiency, which is similar to type 2 diabetes model^21^. Based on the findings of Wan et al., it is possible that PDLIM1 expression is increased in type 1 diabetes mellitus, and endothelial cell function and retinal exudation are attenuated after knockdown of PDLIM1. In comparison, in the type 2 diabetes model we studied, it was found that the expression of PDLIM1 was down-regulated, and the invasion and migration of cells were enhanced after PDLIM1 decreased. These results suggested that PDLIM1 showed a protective effect in diabetic retinopathy caused by type 2 diabetes. Further research is needed to confirm the possible disparity. Based on our previous findings that Wnt3a levels in PDR patients were significantly increased^16^, we hypothesized that PDLIM1 might also play a certain role by regulating the Wnt pathway in DR. Through semi-quantitative analysis, it was shown that Wnt3a expression increased in DR compared to controls. In in vitro experiments, we found that the expression of Wnt3a increased significantly after PDLIM1 knockdown, with enhanced cell apoptosis, migration and invasion ability. These changes were most significant in siPDLIM1 + HG/HL group, which may be induced by further decreased PDLIM1 in RCECs in a high glucose environment. It has been shown that the Wnt pathway is abnormally activated and upregulated in the pathological process of DR. The symptoms of vascular inflammation, leakage and neovascularization in DR can be improved by blocking the Wnt pathway^22,23^. Taken together, our findings suggest that PDLIM1 may reduce the ability of apoptosis, invasion, and migration of RCECs by down-regulating the Wnt pathway and play a protective role in retinal vessels in DR. Further studies are required to confirm these results.

There are some limitations in our study. We did not observe the changes of DR after overexpression of PDLIM1. Moreover, in vivo experiments are required to further verify the role of PDLIM1 in DR. Furthermore, Wan et al.^15^ found a different expression trend of PDLIM1 in DR models, inconsistent with our results. Therefore, further studies in different DR animal models and different periods of DR models are required in the future.

In summary, our results suggest that the expression of PDLIM1 is downregulated in DR, promoting apoptosis, migration, and invasion of RCECs by negatively regulating Wnt3a. PDLIM1 may be a novel biomarker and therapeutic target for DR.

## Supplementary Information

Supplementary Information 1.

Supplementary Information 2.

Supplementary Information 3.

Supplementary Information 4.

Supplementary Information 5.

Supplementary Information 6.

## Data Availability

All data generated or analysed during this study are included in this published article [and its Supplementary information files].

## References

[1] Zheng Y, Ley SH, Hu FB (2018). Global aetiology and epidemiology of type 2 diabetes mellitus and its complications. Nat. Rev. Endocrinol..

[2] Leley SP, Ciulla TA, Bhatwadekar AD (2021). Diabetic retinopathy in the aging population: A perspective of pathogenesis and treatment. Clin. Interv. Aging..

[3] Hendrick AM, Gibson MV, Kulshreshtha A (2015). Diabetic retinopathy. Prim. Care..

[4] Couturier A, Rey PA, Erginay A, Lavia C, Bonnin S, Dupas B, Gaudric A, Tadayoni R (2019). Widefield OCT-angiography and fluorescein angiography assessments of nonperfusion in diabetic retinopathy and edema treated with anti-vascular endothelial growth factor. Ophthalmology.

[5] Campochiaro PA, Akhlaq A (2021). Sustained suppression of VEGF for treatment of retinal/choroidal vascular diseases. Prog. Retin. Eye Res..

[6] Wong TY, Cheung CM, Larsen M, Sharma S, Simó R (2016). Diabetic retinopathy. Nat. Rev. Dis. Primers.

[7] Borrelli E, Grosso D, Barresi C, Lari G, Sacconi R, Senni C, Querques L, Bandello F, Querques G (2022). Long-term visual outcomes and morphologic biomarkers of vision loss in eyes with diabetic macular edema treated with anti-VEGF therapy. Am. J. Ophthalmol..

[8] Sikorska M, Krężel A, Otlewski J (2012). Femtomolar Zn^2+^ affinity of LIM domain of PDLIM1 protein uncovers crucial contribution of protein-protein interactions to protein stability. J. Inorg. Biochem..

[9] Wang H, Harrison-Shostak DC, Lemasters JJ, Herman B (1995). Cloning of a rat cDNA encoding a novel LIM domain protein with high homology to rat RIL. Gene.

[10] Bauer K, Kratzer M, Otte M, de Quintana KL, Hagmann J, Arnold GJ, Eckerskorn C, Lottspeich F, Siess W (2000). Human CLP36, a PDZ-domain and LIM-domain protein, binds to alpha-actinin-1 and associates with actin filaments and stress fibers in activated platelets and endothelial cells. Blood.

[11] Gupta P, Suman S, Mishra M, Mishra S, Srivastava N, Kumar V, Singh PK, Shukla Y (2016). Autoantibodies against TYMS and PDLIM1 proteins detected as circulatory signatures in Indian breast cancer patients. Proteomics Clin. Appl..

[12] Qiu C, Duan Y, Wang B, Shi J, Wang P, Ye H, Dai L, Zhang J, Wang X (2021). Serum anti-PDLIM1 autoantibody as diagnostic marker in ovarian cancer. Front. Immunol..

[13] Li LM, Luo FJ, Song X (2020). MicroRNA-370-3p inhibits cell proliferation and induces chronic myelogenous leukaemia cell apoptosis by suppressing PDLIM1/Wnt/β-catenin signaling. Neoplasma.

[14] Chen HN, Yuan K, Xie N, Wang K, Huang Z, Chen Y, Dou Q, Wu M, Nice EC, Zhou ZG, Huang C (2016). PDLIM1 stabilizes the E-cadherin/β-catenin complex to prevent epithelial-mesenchymal transition and metastatic potential of colorectal cancer cells. Cancer Res..

[15] Wan W, Long Y, Jin X, Li Q, Wan W, Liu H, Zhu Y (2021). Protective role of microRNA-200a in diabetic retinopathy through downregulation of PDLIM1. J. Inflamm. Res..

[16] Gao X, Ma K, Lu N, Hong T, Xu Y, Peng X (2016). Correlation of increased intravitreous WNT3A with vascular endothelial growth factor in proliferative diabetic retinopathy. Retina.

[17] Chen X, Yang K, Sun P, Zhao R, Liu B, Lu P (2021). Exercise improves bone formation by upregulating the Wnt3a/β-catenin signalling pathway in type 2 diabetic mice. Diabetol. Metab. Syndr..

[18] Ríos H, Paganelli AR, Fosser NS (2020). The role of PDLIM1, a PDZ-LIM domain protein, at the ribbon synapses in the chicken retina. J. Comp. Neurol..

[19] Goyal SN, Reddy NM, Patil KR, Nakhate KT, Ojha S, Patil CR, Agrawal YO (2016). Challenges and issues with streptozotocin-induced diabetes—A clinically relevant animal model to understand the diabetes pathogenesis and evaluate therapeutics. Chem. Biol. Interact..

[20] Namekawa, T., Ikeda, K., Horie-Inoue, K. & Inoue, S. Application of prostate cancer models for preclinical study: Advantages and limitations of cell lines, patient-derived xenografts, and three-dimensional culture of patient-derived cells. *Cells*. **8**(1), 74. 10.3390/cells8010074 (2019) 10.3390/cells8010074 PMC6357050 30669516

[21] Fellmann L, Nascimento AR, Tibiriça E, Bousquet P (2013). Murine models for pharmacological studies of the metabolic syndrome. Pharmacol. Ther..

[22] Sheetu BT, Sehgal A, Singh S, Sharma N, Bhatia S, Al-Harassi A, Bungau S, Mostafavi E (2022). Possible role of Wnt signaling pathway in diabetic retinopathy. Curr. Drug Targets..

[23] Yan M, Wang H, Gu Y, Li X, Tao L, Lu P (2021). Melatonin exerts protective effects on diabetic retinopathy via inhibition of Wnt/β-catenin pathway as revealed by quantitative proteomics. Exp. Eye Res..

