# Supplementary figures and images for: PDLIM1 inhibits cell migration and invasion in diabetic retinopathy via negatively regulating Wnt3a
Source: Sci Rep. 2023 Apr 10;13:5820. doi: 10.1038/s41598-023-33073-7 (PMC10086015; doi:10.1038/s41598-023-33073-7)

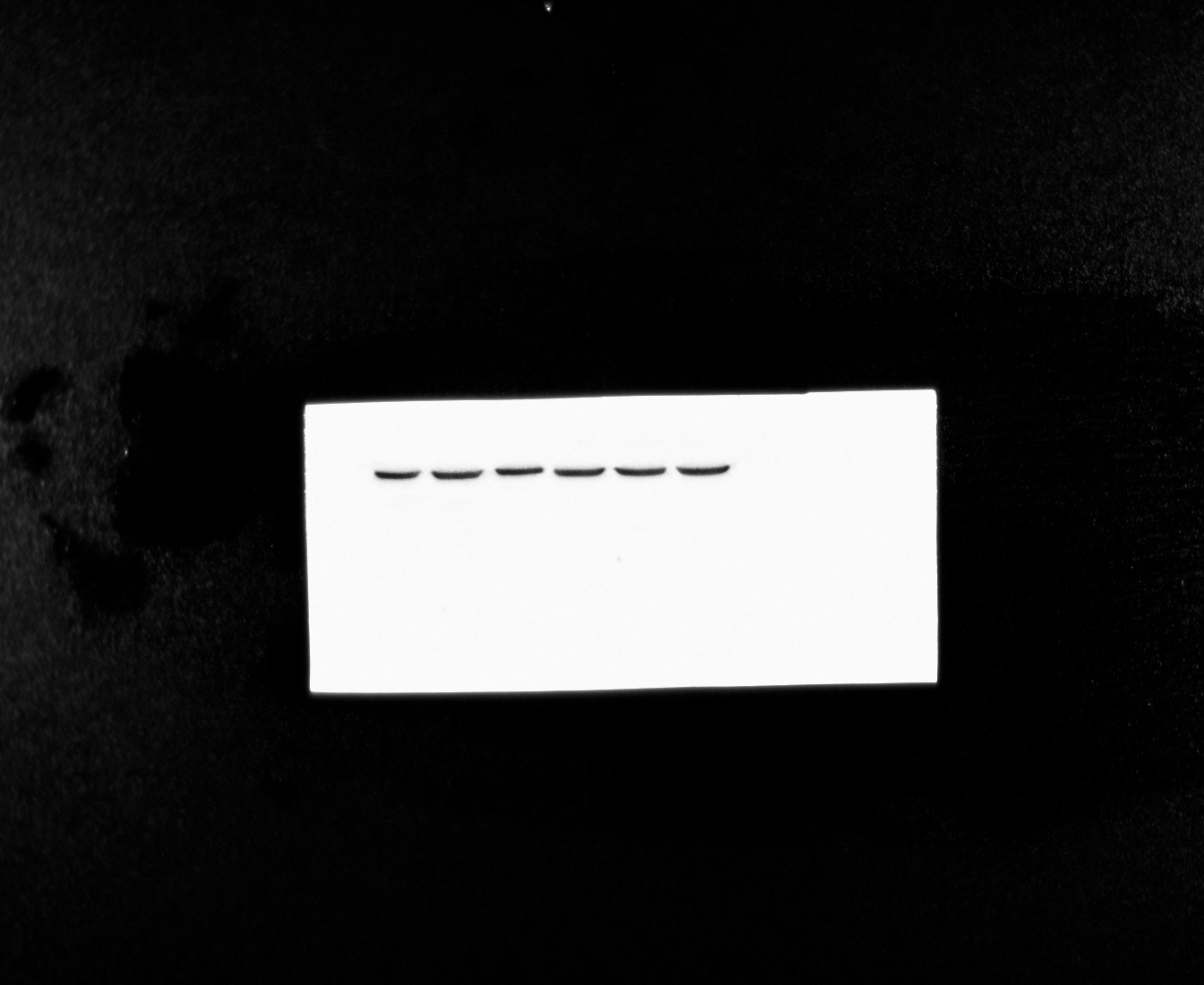

Supplement: Supplementary file 1 — Supplementary Information 1. [file 41598_2023_33073_MOESM1_ESM.tif]

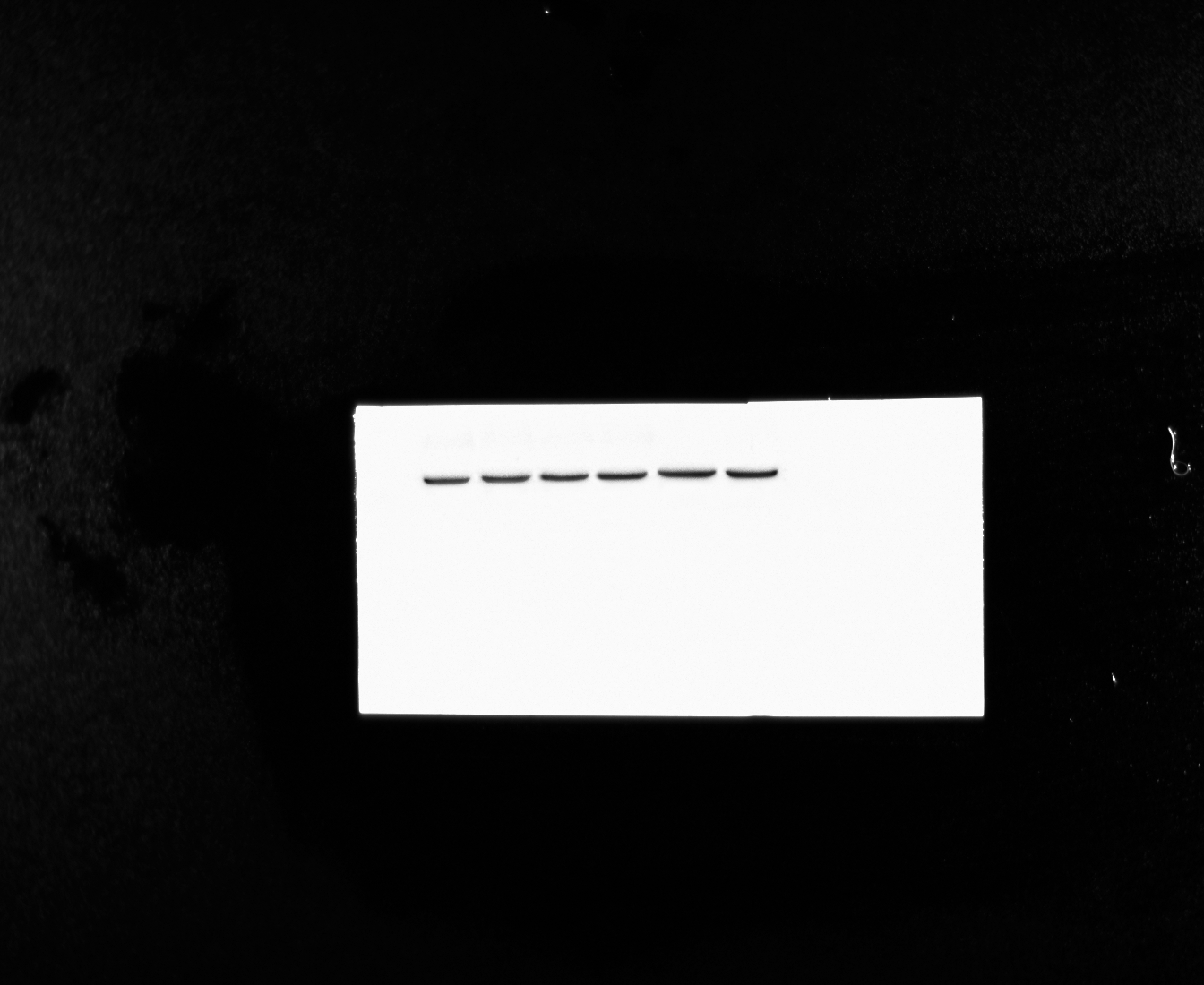

Supplement: Supplementary file 2 — Supplementary Information 2. [file 41598_2023_33073_MOESM2_ESM.tif]

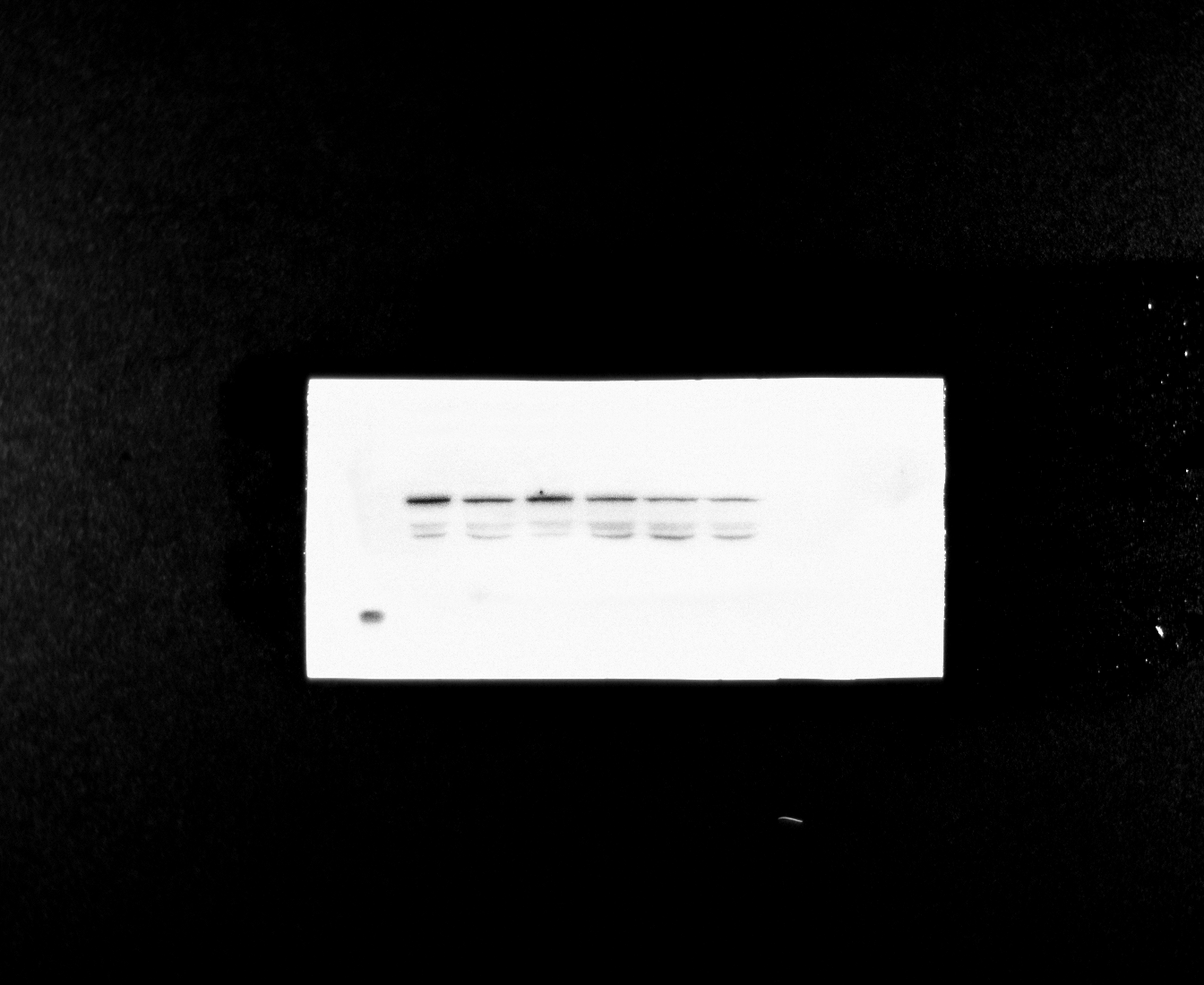

Supplement: Supplementary file 3 — Supplementary Information 3. [file 41598_2023_33073_MOESM3_ESM.tif]

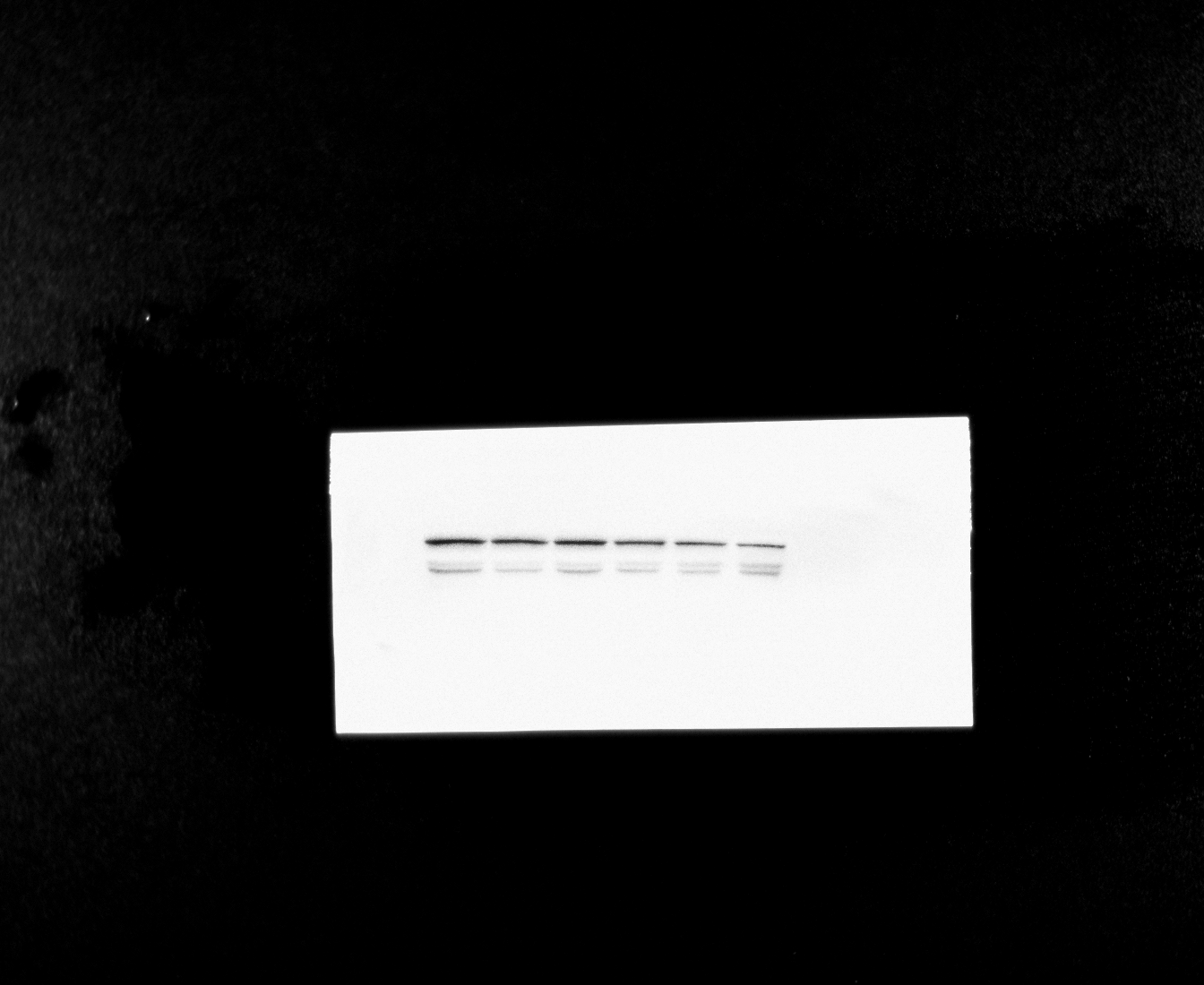

Supplement: Supplementary file 4 — Supplementary Information 4. [file 41598_2023_33073_MOESM4_ESM.tif]

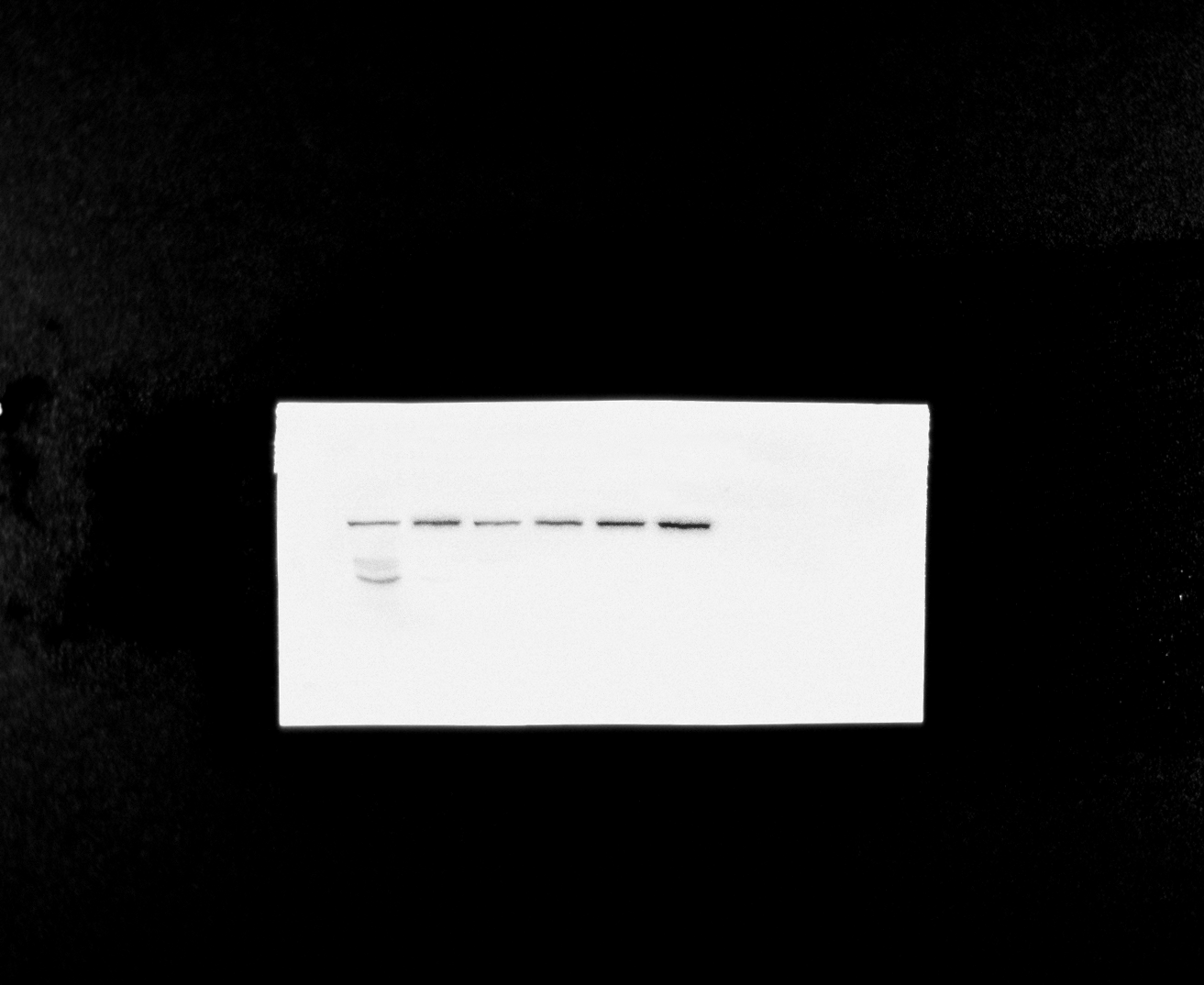

Supplement: Supplementary file 5 — Supplementary Information 5. [file 41598_2023_33073_MOESM5_ESM.tif]

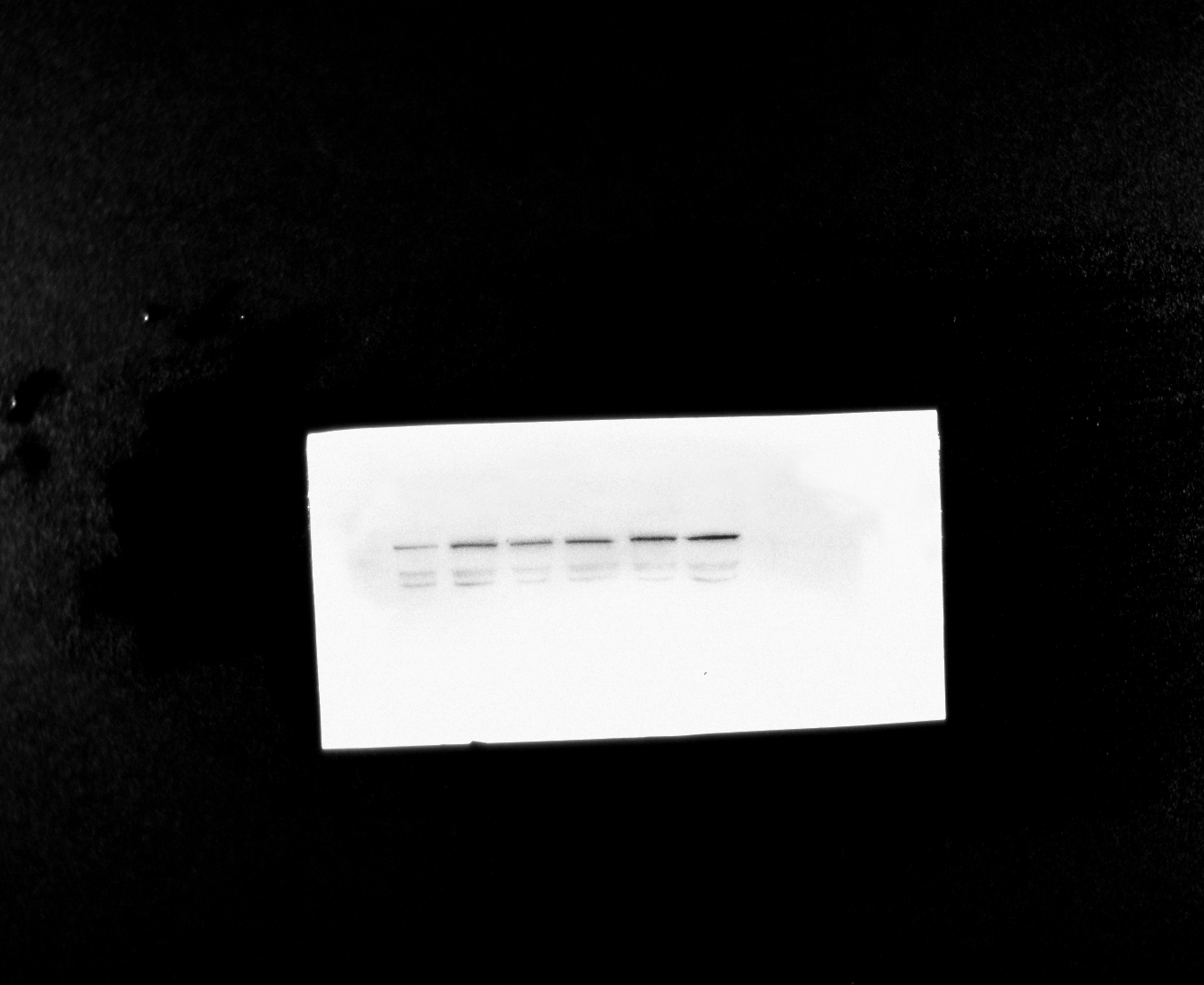

Supplement: Supplementary file 6 — Supplementary Information 6. [file 41598_2023_33073_MOESM6_ESM.tif]
